# The Signaling Petri Net-Based Simulator: A Non-Parametric Strategy for Characterizing the Dynamics of Cell-Specific Signaling Networks

**DOI:** 10.1371/journal.pcbi.1000005

**Published:** 2008-02-29

**Authors:** Derek Ruths, Melissa Muller, Jen-Te Tseng, Luay Nakhleh, Prahlad T. Ram

**Affiliations:** 1 Department of Computer Science, Rice University, Houston, Texas, United States of America; 2 Department of Systems Biology, University of Texas M. D. Anderson Cancer Center, Houston, Texas, United States of America; The University of Tokyo, Japan

## Abstract

Reconstructing cellular signaling networks and understanding how they work are major endeavors in cell biology. The scale and complexity of these networks, however, render their analysis using experimental biology approaches alone very challenging. As a result, computational methods have been developed and combined with experimental biology approaches, producing powerful tools for the analysis of these networks. These computational methods mostly fall on either end of a spectrum of model parameterization. On one end is a class of structural network analysis methods; these typically use the network connectivity alone to generate hypotheses about global properties. On the other end is a class of dynamic network analysis methods; these use, in addition to the connectivity, kinetic parameters of the biochemical reactions to predict the network's dynamic behavior. These predictions provide detailed insights into the properties that determine aspects of the network's structure and behavior. However, the difficulty of obtaining numerical values of kinetic parameters is widely recognized to limit the applicability of this latter class of methods.

Several researchers have observed that the connectivity of a network alone can provide significant insights into its dynamics. Motivated by this fundamental observation, we present the signaling Petri net, a non-parametric model of cellular signaling networks, and the signaling Petri net-based simulator, a Petri net execution strategy for characterizing the dynamics of signal flow through a signaling network using token distribution and sampling. The result is a very fast method, which can analyze large-scale networks, and provide insights into the trends of molecules' activity-levels in response to an external stimulus, based solely on the network's connectivity.

We have implemented the signaling Petri net-based simulator in the PathwayOracle toolkit, which is publicly available at http://bioinfo.cs.rice.edu/pathwayoracle. Using this method, we studied a MAPK1,2 and AKT signaling network downstream from EGFR in two breast tumor cell lines. We analyzed, both experimentally and computationally, the activity level of several molecules in response to a targeted manipulation of TSC2 and mTOR-Raptor. The results from our method agreed with experimental results in greater than 90% of the cases considered, and in those where they did not agree, our approach provided valuable insights into discrepancies between known network connectivities and experimental observations.

## Author Summary

Many cellular behaviors including growth, differentiation, and movement are influenced by external stimuli. Such external stimuli are obtained, processed, and carried to the nucleus by the signaling network—a dense network of cellular biochemical reactions. Beyond being interesting for their role in directing cellular behavior, deleterious changes in a cell's signaling network can alter a cell's responses to external stimuli, giving rise to devastating diseases such as cancer. As a result, building accurate mathematical and computational models of cellular signaling networks is a major endeavor in biology. The scale and complexity of these networks render them difficult to analyze by experimental techniques alone, which has led to the development of computational analysis methods. In this paper, we present a novel computational simulation technique that can provide qualitatively accurate predictions of the behavior of a cellular signaling network without requiring detailed knowledge of the signaling network's parameters. Our approach makes use of recent discoveries that network structure alone can determine many aspects of a network's dynamics. When compared against experimental results, our method correctly predicted 90% of the cases considered. In those where it did not agree, our approach provided valuable insights into discrepancies between known network structure and experimental observations.

## Introduction

Signaling networks are complex, interdependent cascades of signals that process extracellular stimuli, received at the plasma membrane of a cell, and funnel them to the nucleus, where they enter the gene regulatory system. These signaling networks underlie how cells communicate with one another, and how they make decisions about their phenotypic changes, such as division, differentiation, and death. Further, malfunction of these networks may alter phenotypic changes that cells are supposed to undergo under normal conditions, and potentially lead to devastating consequences on the organism. For example, altered cellular signaling networks can give rise to the oncogenic properties of cancer cells [1],[2], increase a person's susceptibility to heart disease [3], and have been shown to be responsible for many other devastating diseases such as congenital abnormalities, metabolic disorders and immunological abnormalities [1],[4].

**Figure 1:**
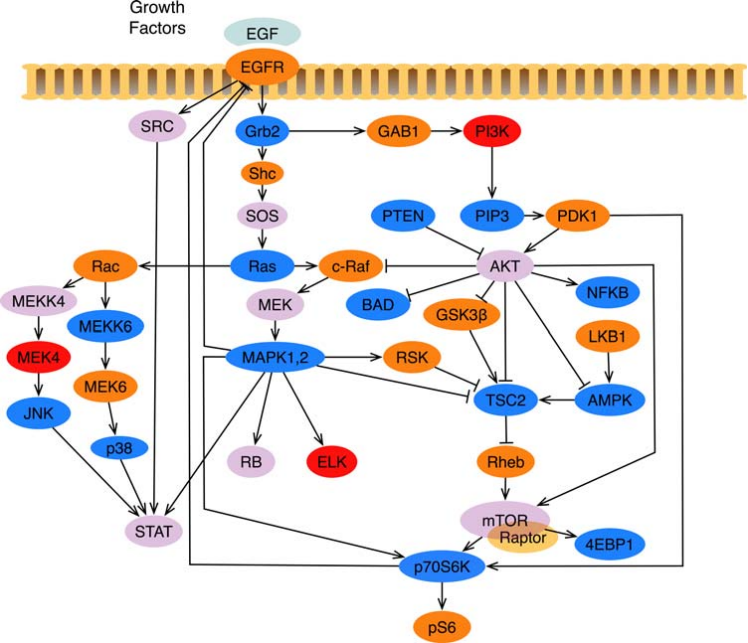
The Model Signaling Network. A MAPK1,2 and AKT network downstream from EGFR, which we assembled from various sources, and used for the case study analysis in this work. An edge from u to v ending with an arrow indicates an activating reaction, while an edge ending with a plunger indicates an inhibiting reaction. With the exception of TSC2, all nodes have self-inhibitory edges, which were added to model the external cellular machinery that regulates the concentration of the active form of the proteins [36]–[43]. Colors were selected to enhance readability of the network.

In light of the crucial role signaling networks play in the proper functioning of cells and biological systems as a whole, and given the grave consequences their alterations may have on the behavior of cells, elucidating the connections in the networks, and understanding how they operate, are two central questions in cell biology. However, unlike the “pathway view” of signaling as linear cascades, signaling networks are highly interconnected, involve cross-talk among several pathways, and contain feedback and feed-forward loops [5]. Figure 1 illustrates this issue in a network of signaling cascades, which is stimulated by EGF and contains several players in cancer pathways. For example, multiple paths lead from EGFR to mTOR-Raptor, resulting in feed-forward loops. Some of these paths activate mTOR-Raptor, while others inhibit it. Further, the network contains two feedback loops, one from p70S6K to EGFR and another from MAPK1,2 to EGFR.

These and other complexities make it very difficult to analyze signaling networks by experimental biology approaches alone. As a result, computational methods have been developed and combined with experimental biology approaches, producing powerful tools for the analysis of these networks [6]. These computational methods produce hypotheses that guide the experimental design, leading to more informative experiments, while experimental results help refine the computational models, resulting in more accurate predictive tools.

In a recent survey, Papin et al. classified existing computational methods into two categories: *structural* and *dynamic* network analysis [6]. Structural network analysis is mainly based on the network's connectivity, which is typically readily available from numerous public signaling network databases (e.g., [7]–[9]), and makes inferences about global network properties as well as individual protein functions. This category can be further refined into two sub-categories, both of which are solely based on connectivity information, yet differ in the type of answers they provide. For example, the methods described in [10]–[13] infer “static” properties of the network, such as numbers of paths, reachability results, etc. In a series of papers, Palsson and co-workers [6], [14]–[16] introduced extreme pathway analysis techniques, which are more appropriate for metabolic networks, yet have been applied to signaling networks to characterize various properties of networks, such as redundancy and cross-talk. Similar analyses have also been formalized and conducted using the principles of S- and T-invariants in Petri Nets (e.g., [17]–[20]).

Methods for dynamic network analysis use, in addition to the network connectivity, the kinetic parameters of the biochemical reactions. The goal of these methods is to model the actual kinetics of the network and obtain through simulation the actual quantities of proteins involved in signal transduction. One of the most widely used techniques in this category is systems of ordinary differential equations (ODEs) (e.g., [21]–[25]). Within such a system, each reaction is modeled by a series of equations connecting reactant concentrations to product concentrations through differential relationships involving reaction rate constants. Given the difficulty of obtaining the numerical values of kinetic parameters [19],[26] and standardization of the parameters and models [27], the applicability of these methods is limited in practice to small-scale networks [6],[28].

Petri Nets have also been used for simulating the dynamics of signaling networks [29]–[31]. While such approaches somewhat relax the necessity for biologically exact kinetic parameters, current Petri Net-based approaches still require the selection of weights and/or probability distributions for individual interactions in the model. As a result, selecting the values for Petri Net parameters presents challenges similar to those encountered in ODE modeling.

Structural network analysis assumes mainly connectivity information about the model, and provides insights into global, static properties of the network. Dynamic analysis in general assumes numerical values of the kinetic parameters, and provides predictions of network dynamics by quantifying the change in concentration and activity-level (the concentration of the active form of a given protein) of the individual proteins and complexes in the network. To obtain a more detailed analysis one must either solve parameter optimization problems for a large number of molecules and interactions or conversely experimentally derive these values.

Given the difficulty of obtaining numerical values of kinetic parameters [19],[26] and the implications this has on the applicability of dynamic analysis methods [6], it is imperative to develop innovative approaches that combine the attractive low requirements of structural network analysis techniques with the detailed answers provided by dynamic analysis techniques—specifically the response of individual proteins to signals which travel through the network.

Several recent efforts in this direction have produced encouraging results. An approach using a boolean network simulation method, based on work in the area of gene regulatory networks, successfully used only signaling network connectivity information to predict the speed of signal transduction through a stomata signaling network [32]. The use of piecewise linear systems of ODEs have also had success in analyzing some of the dynamics of gene regulatory and signaling networks without using exact kinetic parameters (e.g., [33]–[35]). The obstacle to extending the method in [32] to model individual protein responses to signal transduction is the boolean model used to discretize the signal as it propagates. In a boolean model, the signal is either present or absent at each node in the network. Such two-state models of signal transduction simplify the underlying biochemistry to the point where it is difficult to model changes in protein concentration more precisely than present or absent. Modeling such gradients of concentration changes and the effects of those changes may be important to predicting individual protein responses, motivating our effort to devise more fine-grained ways to model and simulate the dynamics of signaling networks. The challenges to using linear-piecewise ODEs to model a signaling network center around the issue of identifying all the ODEs required to model the underlying network as well as scalability issues involved in simulating large systems of ODEs.

In this paper, we extend the synchronized Petri net model and firing policy such that the resulting framework models cellular signaling processes. We call this extension the signaling Petri net (SPN). By coupling this with a novel strategy for Petri net execution and sampling, we obtain a method capable of characterizing some dynamics of signaling networks while using only connectivity information about these networks.

To validate our method, we studied the MAPK1,2 and AKT network shown in Figure 1 in two breast cancer cell lines. This network was chosen because the EGFR receptor and its downstream signaling network play a very important role in development, differentiation, and oncogenic transformation. Two very important signaling molecules within the cell are MAPK and AKT, both of which can be activated by EGFR, and contains several potential regulatory paths between them. We constructed a model network of EGF regulation of MAPK and AKT which includes several feedback and feed-forward loops all of which were constructed based on experimental findings from different laboratories around the world [36]–[43]. We analyzed, both experimentally and computationally, the change in activity-level of several proteins in response to targeted manipulation of TSC2 and mTOR-Raptor. Using the model network, the predictions from our method agreed with experimental results in over 90% of the cases, and in those where they did not agree, our method correctly identified discrepancies that could be traced back to incompleteness in the network connectivity model.

## Materials and Methods

Our approach combines elements of the boolean network simulator in [18] with a synchronized Petri net model [44]. In [18], Li et al. present a non-parametric approach that accurately predicts the speed of signal propagation through a network. However, as their method assumes a binary model of activation—every protein is either active (*true*) or inactive (*false*)—modeling a range of activity-levels is difficult. Petri nets, while able to model concentrations using tokens, require parameters describing the kinetic characteristics of the network, which are typically difficult to obtain.

Our method models signal flow as the pattern of token accumulation and dissipation within places (proteins) over time in the Petri net. Transitions in the network represent directed protein interactions; each transition models the effect of a source protein on a target protein. Through transition firings, the source can influence the number of tokens assigned to the target, called the *token-count*, modeling the way that signals propagate through protein interactions in cellular signaling networks.

In order to overcome the issue of modeling reaction rates in the network, signaling dynamics are simulated by executing the signaling Petri net (SPN) for a set number of steps (called a *run*) multiple times, each time beginning at the same initial marking. For each run, the individual signaling rates are simulated via generation of random orders of transition firings (interaction occurrences). When the results of a large enough number of runs are averaged together, we find that the series of token-counts correlate with experimentally measured changes in the activity-levels of individual proteins in the underlying signaling network. In essence, the tokenized activity-levels computed by our method should be taken as abstract quantities whose changes over time correlate to changes that occur in the amounts of active proteins present in the cell. It is worth noting that some of the most widely used experimental techniques for protein quantification—western blots and microarrays—also yield results that are treated as indications, but not exact measurements, of protein activity-levels within the cell. Thus in some respects, the predictions returned by our SPN-based simulator can be interpreted like the results of a western blot or microarray experiment looking at changes relative to “control”.

The key insight behind our approach is the assumption that, while all network parameters determine the actual signal propagation to some extent, the network connectivity is the most significant single determinant. While this is clearly a gross simplification, several researchers have observed that the connectivity of a biological network dictates, to a great extent, the network's dynamics [18], [45]–[47]. Some have conjectured that biological network connectivities have evolved to have a stabilizing effect on the overall network behavior, making the network more resilient to local fluctuations in other network parameters such as reaction rates and protein binding affinities [45],[47]. Here we present the *signaling Petri net* (SPN) model and the signaling Petri net-based simulator whose designs collectively utilize this assumption and couple it with a Petri net tokenization scheme that quantifies the changes in protein activity-levels that occur as signals propagate through the network. In the following sections, we describe the synchronized Petri net, how we extended it to create the signaling Petri net, and a novel strategy for executing the signaling Petri net to simulate signaling network dynamics.

### Petri Nets

A Petri net is a graph that consists of two types of nodes, *places*, and *transitions* [44]. Edges in the graph, called *arcs*, are directed and connect places to transitions or transitions to places. Thus, the Petri net is a bipartite graph. Formally, a Petri net is a 4-tuple *Q* = 〈*P*,*T*,*I*,*O*〉 where

1. *P* = {*p*_1_,*p*_2_,…,*p_m_*} is the set of places,
2. *T* = {*t*_1_,*t*_2_,…,*t_n_*} is the set of transitions,
3. *I* = {*i*_1_,*i*_2_,…,*i_k_*} is the set of input arcs where for all (*u*,*v*)∈*I*, *u*∈*P* and *v*∈*T*, and
4. *O* = {*o*_1_,*o*_2_,…,*o_l_*} is the set of output arcs where for all (*u*,*v*)∈*I*, *u*∈*T* and *v*∈*P*.

In order to simulate a dynamic process, a number of tokens is assigned to each place in order to indicate the presence of some quantitative property. This assignment of tokens to places encodes the state of the system and is called a marking, denoted **m**. A *marked Petri net*, *R* = 〈*Q*,**m**_0_〉, is a Petri net with a marking **m**_0_, called the initial marking. For the remainder of this paper, the term *Petri net* (PN) refers to a marked Petri net.

Changes in the state of the system are simulated by *executing* the Petri net—evaluating the effect of transitions on the marking of the network. These changes in marking are induced by sequential *firing* one or more transitions. When a transition fires, it removes a token from each place connected to it by input arcs and adds a token to each place connected to it by output arcs. The number of tokens removed from inputs and added to outputs can be specified by weighting the input arcs. However, as our extension does not use this weighting property, we do not consider this very common PN formulation here.

A transition can only fire when it is *enabled*, meaning that each of its input places has at least one token in the current marking. If a transition *t*, when fired on a marking **m**_1_, produces marking **m**_2_, then we write **m**_1_|*t*〉**m**_2_.

This notation can be extended to represent the effect of firing a series of transitions. A *firing sequence*, σ = (*t*_1_,*t*_2_,…,*t_j_*) is a sequence of transitions. The sequence's cumulative effect on the system's state is denoted **m**_0_|σ〉**m** *_f_* where **m**_0_ is the initial marking and **m** *_f_* is the marking produced by the firing of the sequence of transitions in the order specified in σ. In this paper, we write 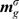 to indicate the marking produced by the first g transitions in σ. Therefore, in the above example, 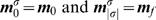.

For a more complete introduction to types of Petri nets and their properties, we refer the reader to [44].

#### Synchronized Petri nets

Synchronized Petri nets model systems in which the firing of a transition is triggered by a specific event that occurs in the environment. The marked Petri net is extended to include a set of these events and a mapping function that assigns an event to each transition. When transition t's assigned event occurs, transition t is fired. Formally, a synchronized Petri net is a 3-tuple 〈*R*,*E*,*Sync*〉, where [44]:

R is a marked Petri net,

*E* = {*e*_1_,*e*_2_,…,*e_s_*} is a set of events, and

*Sync*:*T*→*E*∪{**e**} maps each transition in the Petri net to an event. Event **e** is the *always occurring event*. Any transition associated with **e** is always immediately fired upon becoming enabled.

When executing a synchronized Petri net, transition t is fired when its associated event *e* = *Sync*(*t*) occurs. The order in which events are generated depends upon the environment which generates them. Just as in the marked Petri net, when a transition fires, it removes one token from each place connected by input arcs and gives one token to each place connected by output arcs.

As will be discussed in the next sections, we extend the synchronized Petri net paradigm to model the dynamics of a signaling network. To our knowledge, ours is the first use of the synchronized Petri net to model biochemical systems. In principle it is well suited to signaling networks since places represent proteins, tokens represent concentrations, and transitions represent directed protein interactions. A model of signaling event occurrence can be used to generate events and fire transitions, providing a way of simulating the signaling network's behavior. These and other design details will be discussed in the next section.

### The Signaling Petri Net-Based Simulator

**Figure 2:**
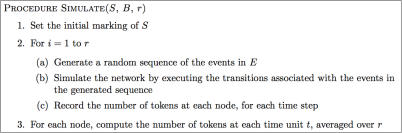
A High-Level Outline of the Procedure for Simulating a Signaling Network. The input to the procedure is a signaling Petri net, S, the number of time units to simulate the network for, B, and the number of runs for which to repeat the simulation, r. The random generation of event ordering is employed to simulate the stochasticity in reaction rates and the differing times of signal arrivals.

A high-level sketch of our simulator is given is Figure 2. Details and rationale for specific design decisions will be discussed in subsequent sections.

During the simulation, the input signaling Petri net is executed multiple times on a firing sequence constructed by the signaling event generator. The signaling event generator imposes an ordering on transition firing such that it creates a two-time scale simulation. The smaller time scale is discretized as the firing of a single transition. This unit is referred to as the *firing* time scale. Firing steps are nested within a larger time scale, called time *blocks*, in which each transition is fired exactly once. Thus, there are |*T*| firings per block. Since the simulation is run for the specified number of time blocks, B, there are *B*|*T*| firing steps in the simulation.

**Figure 3:**
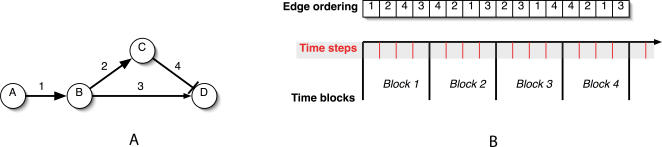
The Effects of Reaction Rates on Signal Propagation. (A) By changing the speed of signaling edge 3, the value of D at the end of a single simulation step can be reversed. If edge 3 is slower than the cascade B→C⊣D, then D will be active. If edge 3 is faster than the cascade, then D will be inactive. (B) An example of how the simulator might evaluate the individual edges during a run. In each time block, every edge is evaluated once. Each edge evaluation corresponds to one time step. Note that the order of the edge evaluation is shuffled during each time block in order to sample the space of possible relative signaling rates.

The time structure for an example simulation is illustrated in Figure 3. This dual-time approach is necessitated by the rate parameter sampling strategy we employ. Since the rate parameters are not known, our method executes many simulation runs (Step 2 in Figure 2) in order to sample the space of possible rate parameters. The markings returned by these runs are then averaged (Step 3 in Figure 2). The only requirement placed on the different rate parameter values is that all events occur within the same larger time frame—the time block. Therefore, within every time block all edges are evaluated once, though not necessarily in the same order.

This idea of evaluating random event orderings within a two-time scale system has appeared before in the domain of transcriptional networks [48]. In that study, Chaves et al. employed a two-time scale formulation of network updates similar in concept to the one we describe here. In their work, they assumed a boolean model of regulation and characterized the effect of different relative rates of transcription within the same network on the final steady state reached. In contrast, our method is designed to operate on tokenized models of signaling networks with the ultimate intent of predicting the activity-level changes of proteins in the underlying signaling network over time.

In the next sections, we discuss in greater detail the core design decisions underlying our method: the signaling Petri net, transition firing, signaling network event generator, constructing the initial marking for the model, and sampling signaling rates. We then discuss how our strategy can be used to predict the outcome of perturbation experiments.

### The Signaling Petri Net

The goal of our method is to predict the signal flow through a cell-specific network under specific experimental conditions. As a result, the signaling Petri net model must characterize the connectivity of the signaling network, the connectivity-level network properties that are unique to the cell type and experimental conditions under which the network is being studied, and the signaling processes of activation and inhibition.

The signaling Petri net is a synchronized Petri net with: 1) a specific way of modeling activating and inhibiting interactions using places, transitions, and arcs; 2) a one-to-one correspondence between events and transitions such that every transition is associated with a unique event; 3) modified rules regarding how many tokens are moved in response to a transition firing; and 4) a signaling network event generator.

**Figure 4:**
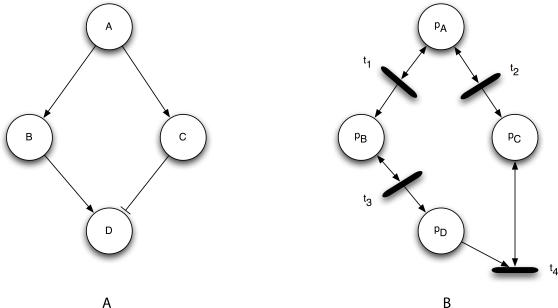
An Example Signaling Network and Its Corresponding Petri Net. An example signaling network (A) and its corresponding Petri net (B). Each signaling protein in the network, A, B, and C, are designated as places p_A_, p_B_, and p_C_. Signaling interactions become a transition node and its input and output arcs. Note that the connectivity for an activating edge differs from that of an inhibitory edge.

Places correspond to the activated forms of signaling proteins. The number of tokens assigned to place p in marking **m** *_s_*, **m** *_s_*(*p*), abstractly represents the amount of active protein p present in that network state. Signaling interactions are modeled using transitions and their connected input and output arcs. Each transition, t, is associated with a unique signaling event, e, such that when e occurs, transition t fires. Figure 4 shows the equivalent signaling Petri net for a signaling network.

Formally, a signaling Petri net is a 3-tuple *S* = 〈*R*,*E*,*Sync*〉, where:

R is a marked Petri net,

E is a set of signaling events such that |*E*| = |*T*| and there is no *always occurring event*, and

*Sync*:*T*→*E* is a one-to-one mapping which assigns each transition a unique signaling event.

The initial marking of a signaling Petri net, **m**_0_, represents the state of rest from which the network is starting and being simulated. Proteins whose concentrations are known to be high can be given a large number of tokens, and those whose concentrations are known to be low can be assigned few or zero tokens. Attention to the initial marking is central to modeling cell-specific networks. In many cell lines, specific proteins are known to contain mutations that render them perpetually active or inactive [49]. Furthermore, experimental studies frequently involve the targeted manipulation of various proteins within the network. Both of these phenomena induce state changes in certain proteins at various time points that must be modeled. The way in which these are modeled will be discussed when the simulator design is explained.

### Transition Firing

When a signaling interaction *A*→*B* (A *activates* B) or A⊣B (A *inhibits* B) occurs, it has the effect of changing the state of the system by modifying the activity-level of A and/or B. Thus, in the SPN used to model this network, the associated transition, t, will fire at time τ and produce marking **m**_τ+1_ from **m**_τ_. The way in which **m**_τ+1_ is computed from **m**_τ_ depends on the set of input and output arcs attached to the transition as well as the number of tokens moved by the transition.

The combination of input and output arcs connected to a transition is determined exclusively by the type of interaction and the transition firing model. However, different topologies, combinations of input and output arcs, are needed to model the different biochemical processes that mediate protein-protein interactions in a signaling network. Here we examine four of the most common biochemical processes, identify the corresponding topological motifs, and ultimately devise a modeling policy best suited for non-parametric simulation of signal flow.

**Figure 5:**
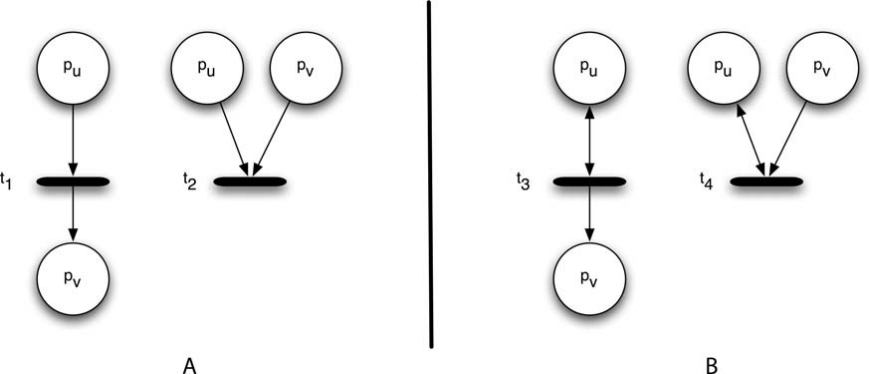
The Topological Motifs for Differing Signaling Processes. (A) The token consumption motifs for complexing and recruitment. Transition t_1_ encodes activation of v by the binding or consumption of u. Transition t_2_ encodes deactivation of v by the binding or consumption of u. In both cases, the number of tokens of p_u_ decreases immediately after transitions t_1_ and t_2_ fire. (B) The token conserving motifs for PTM and GTP/ATP binding. Transition t_3_ encodes enzymatic activation of v by u. Transition t_4_ encodes enzymatic inhibition of v by u. In both cases, the number of tokens of p_u_ remains unchanged immediately after transitions t_3_ and t_4_ fire.

In *post-translational modification* (PTM), a protein mediates the addition or removal of a phospho group at a specific phosphorylation site on another protein. In *GTP/ATP* binding, a protein triggers the exchange of GDP (ADP) from GTP (ATP) on another protein. In a *recruitment* process, a protein mediates the relocalization of another protein to a different part of the cell. Finally, in a *complexing* process, a protein binds to another protein to create a complex, which can then participate in other reactions. In the first two processes, the mediating protein usually acts as an enzyme that participates in the reaction but is not consumed by the reaction. In the latter two processes, the participating protein often becomes unavailable to other reactions, transiently while the protein recruitment is taking place and for longer durations when complexing occurs. To model these two cases, we identified the two different token-passing policies implemented by the different topological motifs depicted in Figure 5.

#### Token consumption

In this policy, uΠv consumes tokens in u in order to generate new tokens for v. In order to model this, p_u_ is connected to transition t_1_ through an arc and p_v_ is connected to t_1_ through an output arc. When t_1_ fires, some number of tokens in p_u_ are moved into p_v_. Similarly, u⊣v consumes tokens in u in order to consume tokens in v. This is modeled by connecting p_u_ to t_2_ with an input arc and p_v_ to t_2_ with an input arc. When t_2_ fires, some number of tokens are removed from both p_u_ and p_v_. This policy models a recruitment or complexing event in which u binds to another molecule, thereby creating a molecule of type v. *A molecule of type u has been consumed in order to generate or deactivate a molecule of type v.*

#### Token conservation

In this policy, uΠv generates new tokens for v while conserving those in u. In order to model this, p_u_ is connected to transition t_3_ through a read arc. Node p_v_ is connected to t_3_ through an output arc. When t_3_ fires, some number of tokens in p_u_ is read (but not removed) and copied into p_v_. Similarly, u⊣v consumes tokens in v while conserving those in u. This is modeled by connecting p_u_ to t_4_ with a read arc and p_v_ to t_4_ with an input arc. When t_4_ fires, some number of tokens in p_u_ are read and removed from p_v_. Enzymes will often behave in this way: inducing a change in a molecule (v) without themselves undergoing any change. *A molecule of u has induced a change in a different molecule of type v without itself changing state.*

Ideally, for each interaction in the network, the associated transition could be embedded in the topology corresponding to the interaction's underlying biochemical mechanism. However, connectivity-level knowledge of the network does not provide this information for each interaction. In the absence of these details, we use one token-passing policy for all interactions in the network. We implemented and tested both the consuming and conserving policies and found that token conservation provides significantly more accurate results when compared to experimentally derived data. This is not surprising, as post-translational modification and GTP/ATP binding events are responsible for many activation state changes in signaling networks [1], [50]–[52]. It is worth noting that our approach does not restrict the net structure to token conserving topologies. Thus, it is possible to use the token consumption topologies where such processes are known to occur. However, as our focus in this paper is designing a purely non-parametric simulation method, we consider the use of information regarding the biological mechanism of signaling as a potential way to further improve the accuracy of our method's predictions and identify this as a direction for future work.

The transition topologies, as described above, do not designate how the number of tokens added to or removed from p_v_ is determined. However, we know that in biochemical signaling networks concentration has an effect on the strength of a signaling event [53]–[55]. Specifically, the higher u's concentration, the stronger its effect on v—the more tokens that p_u_ has, the more tokens of p_v_ should be affected (generated or consumed).

However, because of the stochastic nature of the underlying biochemistry, it would be inaccurate to assume that *all* active u molecules will always participate in an interaction with v. In order to accommodate this observation, when transition t fires, we randomly select the number of p_u_'s tokens to be involved in the subsequent evaluation of the transition, which we call a *signaling event*. Note that, according to our choice of topology, p_u_ can always be identified as the node connected to the transition by a read arc. In this paper, we assume a uniform distribution for selecting the number of tokens involved in a given signaling event, but acknowledge that other distributions may be more appropriate under certain circumstances and identify this as a topic deserving further consideration.

Let *m_s_*(*x*) denote the number of tokens in node x at time s. For an interaction (u,v), under the token conservation policy detailed above, u's token-count remains unchanged after the firing of t, whereas v's token-count is updated based on the following formula:
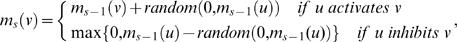
where *random*(p,q) is a random integer drawn from a uniform distribution over the range [p,q].

If we employ the policy of token passing with consumption, then after *m_s_*(*v*) has been computed based on the formula above, *m_s_*(*u*) is updated as:
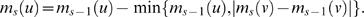


### Signaling Network Event Generator

The SPN topology and transition token-number selection policy alone do not specify the speed with which individual signaling interactions occur. However, such rates must be accounted for when simulating a signaling network. ODEs characteristically model such details as reaction rate constants; parameterized Petri nets specify these in a variety of ways including transition firing rates and firing probabilities [17],[30]. In synchronized Petri nets, the environment controls the generation of events. Thus, the signaling network event generator is responsible for controlling the timing and ordering of signaling events. However, as our objective is a non-parametric simulation method, our approach must either estimate these parameters or operate without explicit knowledge of them.

Estimating reaction rates using only connectivity is currently beyond the predictive or inferential capabilities of computers. While there has been some work in the area of predicting reaction rates, all results of which we are aware require knowledge about the mechanism of signaling (e.g., [56]). As a result, without enriching the SPN model, it is doubtful that rate parameters can be accurately estimated.

For this reason, the signaling network event generator operates without explicit knowledge of the rate parameters. To compensate for this “missing” knowledge, we make use of an observation of signaling networks discussed earlier: a network's connectivity determines its dynamics. Several studies have found that the connectivity of biochemical networks desensitizes them to small fluctuations in the kinetic biochemical parameters [45]–[47]. Understood within the context of evolution – a stochastic process that tweaks signaling network parameters across generations – this is a highly desirable property as it ensures that an offspring remains viable despite fluctuations in the exact tuning of its cellular machinery. If this property holds, then small fluctuations in the rate parameters should have a marginal effect on the overall propagation of signal through the network. We can consider these small effects to be noise obscuring the underlying dynamics of the network connectivity. By taking many samples of the network dynamics under a variety of reaction rate assignments and then averaging these dynamics, we simultaneously reduce the noise introduced by any one rate assignment and strengthen the underlying dynamic characteristics of the network's connectivity.

However, since reaction rate constants can vary by several orders of magnitude—from 10^−10^ to 10^3^, the task of correctly selecting parameters *close* to the true parameters is non-trivial. In fact, without having some estimate of the actual rate parameters, it is unclear as to how to measure closeness at all. Clearly, these are among the issues that make parameter estimation so difficult for ODE and Petri net approaches. Since our comparisons will be relative and not absolute, we take a relative approach to modeling rate parameters. The space of possible rate values is *the space of possible signaling event orderings*.

This idea is illustrated in Figure 3A. Protein A affects the activity of protein D through two separate pathways. Assuming that A is active to begin with, the relative speed of these two pathways determines the final activity of D. If the pathway through C is faster than the pathway BΠD, then D will be active. However, if the pathway speeds are reversed, then D will remain inactive. The overall outcome of this network can be represented without any use of numeric reaction rates by representing the reaction rates as an ordering over all the edges in the network. We can extend this idea to the SPN by observing that there exists a unique event for each signaling edge in the signaling network.

**Figure 6:**
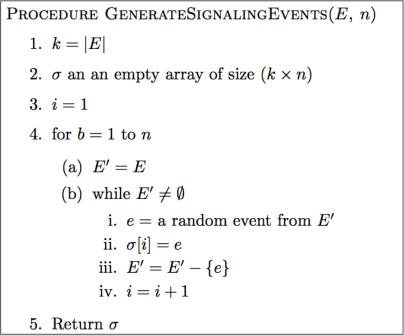
The Algorithm That Implements the Signaling Network Event Generator. This routine generates the time block/firing structure. Given a set of events, E, and the number of blocks for which the SPN will be executed, n, GenerateSignalingEvents generates n blocks of events, each consisting of |*E*| events ordered randomly. In each block, every event in E occurs exactly once.

This sampling strategy is the motivation for the dual-time framework depicted in Figure 3B and implemented by the signaling network event generator shown in Figure 6. *Time blocks* are the larger time intervals during which every signaling event occurs exactly once. Since every transition in the SPN is associated with a unique event, each transition will fire exactly once in each time block. *Transition firings* are the smaller time units that impose a strict sequential order on the occurrence of signaling events. While this strict sequentiality of firing models relative reaction rates, it also discretizes the effect of signaling events. Though this is consistent with the definition of transition firing in discrete time Petri nets (only one transition is evaluated at a given point in time) [44], in biological signaling networks there is no such serial evaluation constraint. However, our validation with experimental data suggests that this discretization approximation does not affect the overall validity of the simulation results.

### Defining the Initial State

As mentioned previously, the initial state of the SPN is the initial marking, **m**_0_. As the SPN provides no explicit information on how this marking should be built, we propose three ways to construct the initial state: zero, basal, or experimentally derived. In a zero initial state, the simulator initializes all proteins to have zero tokens. The basal initial state is a random distribution of activation levels intended to model the cell when no impulses due directly to external stimuli are propagating through the signaling network. Though a basal network is considered at rest, in general it will not have a zero marking since signal flows are known to occur even in unstimulated signaling networks through autocrine and paracrine secretions by the cells. The experimentally derived initial state is based on knowledge about the activity levels of various proteins just prior to the addition of the external stimuli.

When accurate experimental data is available such as results from microarrays or western blots, the experimentally derived initial state may be the most accurate. A challenge in using experimental data, however, is determining how best to assign numbers of tokens based on the experimentally observed activity levels.

In the absence of reliable experimental data, the basal initial state seems more accurate than the zero initial state. However, it presents the challenge of properly selecting the basal activity-levels to assign to each protein in the model network. In [18], a basal initial state was constructed by activating a small number of randomly selected proteins in the signaling network. However, the work in [18] was done using a boolean model. Translating this approach into a tokenized model creates the additional complexity of determining how many tokens each basally active protein should receive. The correct values are likely to depend on the specific signaling network and experimental conditions.

We performed preliminary tests to compare the effect of using different basal versus zero markings on the outcome of the simulator. We found that the basal and zero states produced indistinguishable predictions so long as less than 30% of the proteins were activated and a small number of tokens (<5) were used when constructing the basal marking. This is not as surprising as it may seem at first. Inhibitory edges will quickly consume a small number of tokens scattered throughout the network, effectively returning much of the network to the zero state before a stimulation event can propagate through.

Furthermore, while validating our method, we also compared the predictions produced by SPNs based on a zero initial state and experimentally derived initial state. These, too, did not produce noticeably different final results for similar reasons as discussed above. Details of these comparisons will be discussed further in the Results and Discussion sections.

However, since all three initial state construction strategies yield qualitatively identical predictions, using zero initial states has the advantage of invoking the fewest unnecessary assumptions about the network (as in the case of the basal initial state) and requiring the least experimental data (as in the case of the experimentally derived state). Nonetheless, in our implementation of the tool, we allow for using any one of these three initial state construction strategies.

### Modeling Cell-Specific Signaling Networks

Whereas consensus signaling networks typically represent the connectivity in normal cells, many experiments are conducted on abnormal cells in which oncogenic mutations, gene knockouts, and pharmacological inhibitors have altered the behavior of various signaling nodes in the network. In an SPN, these alterations to the signaling network can be modeled by adding/removing transitions (and associated input/output arcs) and explicitly setting the token count for various proteins in the initial state.

The two network alterations which are commonly induced by oncogenic mutations, gene knockouts, or pharmacological inhibitors are constitutively high or low protein activity-levels, meaning that a protein is either unable to be inhibited or unable to be activated. The simulator allows for proteins to be specified as either fixed *High* or *Low*. Here we explain how these are modeled by changes to the SPN.

If protein u is fixed high, then this protein cannot be inhibited. Thus, all transitions that remove tokens from p_u_ are removed from the SPN. The fact that u is high, however, also suggests that it maintains a higher activity level in general. Therefore, in the initial state, **m**_0_(*p_u_*) = *H*, where H is a non-zero number of tokens. Since all inhibiting transitions have been removed from the SPN, throughout any execution, place p_u_ will always have at least H tokens.

In experiments, we have observed that the choice of the value of H does not change the relative outcome of the simulations. While H will affect the actual number of tokens present in a given place as well as the number of time blocks required to observe certain activity-level changes, the relative changes in activity-level (number of tokens) among different proteins (places) does not change. As a result, one is free to select any reasonable value of H (for our experiments, we used H = 10) as long as this H is held constant across all simulations whose results will be compared.

If protein u is fixed low, then this protein cannot be activated. Thus, all transitions that add tokens to p_u_ are removed from the SPN. The fact that u is low, however, also suggests that it maintains a constantly low activity level in general. Therefore, in the initial state, **m**_0_(*p_u_*) = *L*, where L is a small number of tokens (in our simulations we use L = 0). Since p_u_ is only inhibited, we observed that all constitutively low proteins quickly had their marking reduced to zero.

Unlike the value of H, extra caution must be taken when selecting values for representing L. A value of L that is too large can destabilize the early propagation of signal through the network. In our experiments, we obtained best results for values of L very close to or equal to zero (*L*≤2). Beyond this, the final results obtained depended on other values in the network, the strength of the signal, and the duration of the simulation.

### Simulating a Signaling Network

**Figure 7:**
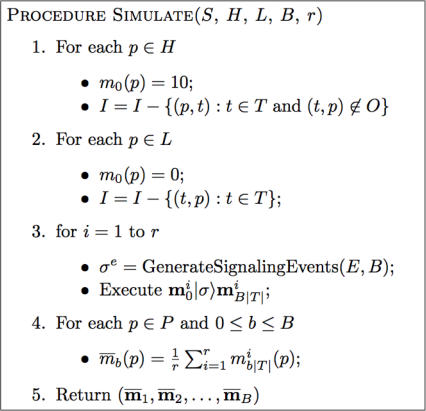
The Procedure for Simulating a Signaling Petri Net. Simulate predicts the signal flow through the SPN S. The simulation is run for B time blocks; the results of r runs are averaged to produce the final result. Most of the work is done by the signaling Petri net execution procedure detailed in the preceding sections. This execution actually performs an individual run. This procedure takes the initial marking, m_0_, and applies the sequence of transitions triggered by the event sequence, σ^e^. This ordering, generated by the algorithm in Figure 6, has the dual time structure in which each block of edges contains every event in E exactly once. Each firing evaluates the effect of one transition. The markings at the end of each time block are extracted in Step 5.

Figure 7 provides more detailed versions of the simulation algorithm outlined in Figure 2. Steps 1 and 2 of the Simulate procedure constructs the initial marking and net topology to incorporate perpetually high proteins, H, and perpetually low proteins, L. In this paper, proteins that are assigned high activity-levels receive an initial token count of 10 in order to model a higher-than-average initial activity-level. As discussed earlier, using other values of H scale the activity-levels of all the proteins in the network, but will not qualitatively change their relative activities.

The loop in Step 3 runs r individual simulation runs. Each run receives a different event ordering, σ^e^, thereby implementing the interaction rate sampling strategy. The time block/step structure is contained within the ordering σ^e^ (see Figure 6C). As a result, the SPN execution step simulates the events by firing their associated transition. Only those markings that correspond to time block boundaries are sampled.

After Simulate finishes collecting the time block markings from all the runs, Step 4 computes the average markings for each time block and Step 5 returns these averages.

### Simulating a Perturbation Experiment

We tested the accuracy and performance of our method by simulating the effect of two different targeted manipulations to a well-known signaling network. We compared these predictions to experimental results produced by performing the actual manipulations on two separate cancer cell lines.

The perturbations we considered in this study altered the constitutive activity-level of various proteins in the network (as opposed to affecting specific signaling interactions). Therefore, we modeled the perturbations as changes in the high and low proteins—H^c^ and L^c^ for the control (unperturbed) network and H^p^ and L^p^ for the perturbed network.

**Figure 8:**
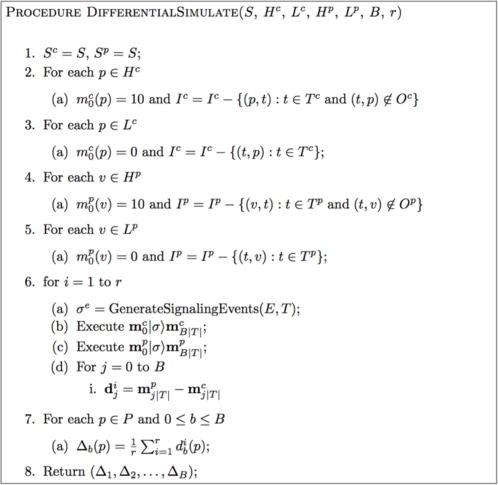
The Algorithm for Predicting the Effect on Signal Propagation of a Targeted Manipulation. The algorithm for predicting the effect on signal propagation of a targeted manipulation on signaling network with connectivity G. The ‘c’ and ‘p’ superscripts are used to denote parameters in the *control* and *perturbed* versions, respectively, of the SPN.

A variant of the Simulate method was required to quantify how a perturbation changed the protein token-counts for each time block. Figure 8 shows the algorithm we used. In the procedure DifferentialSimulate, the input S provides the consensus SPN. Inputs H^c^ and L^c^ specify the control high and low proteins, the inputs H^p^ and L^p^ specify the perturbed high and low proteins. After Steps 1–5 construct two separate SPNs for the control and perturbed conditions, the loop in Step 6 performs r independent simulations over the control and perturbed models. Step 6d computes the difference between the markings at the end of each time block in the perturbed and control networks. The marking difference 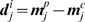 yields the marking 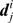 where 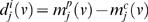 for each *v*∈*P*. Following the loop, the marking differences are averaged to obtain the time series (Δ_1_,Δ_2_,…,Δ*_B_*) where Δ*_b_*(*v*) is the average change in the token-count for protein v at time block b.

For values of |Δ*_b_*|>0 for a given molecule v, we can conclude that the perturbation caused a change in the activity-level of v at time block b only if the difference observed is statistically significant. We use a t-test to determine whether this change is statistically significant for protein v at time block b. Computing the t-test for two distributions (control and perturbation) requires knowledge of the mean (μ*_c_*_,*b*_ and μ*_p_*_,*b*_) as well as the variance 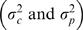 for both distributions. In order to obtain these parameters for the control network, a large number, X, of independent simulations is run. Simulation i provides a single series of markings, 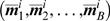. The mean is then computed:
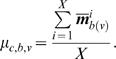
The variance is computed similarly:
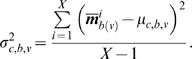
The parameters μ*_p_*_,*b*,*v*_ and 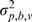 for the perturbed network are computed as described above by substituting the perturbed network for the control network. Using these parameters, the t-value for molecule v at time block b can be computed from the formula
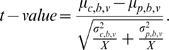
The statistical significance of the difference can then be obtained by comparing the t-value to the desired critical value.

Note that the DifferentialSimulate procedure and the associated significance test can predict the effect not only of perturbations, but also of any two different experimental (or cellular) conditions imposed on the same signaling network. As a result, in addition to perturbation experiments, our method can also be used to study the effects of other phenomena that induce changes in the propagation of signal through a signaling network.

## 

### Cell-Specific Signaling Network Models

Figure 1 shows the signaling network we analyzed. We obtained the core connectivity from a published literature survey on the EGFR network [57]. We added to this several other well-established interactions taken from literature [36]–[43]. The response of this network to various perturbations was measured and simulated in two separate breast cancer cell lines: MDA231 and BT549. The core signaling Petri net used, S^EGFR^, is captured by the following signaling proteins and interactions: places (the set P): v_EGFR_, v_SRC_, v_Rac_, v_MEKK4_, v_MEK4_, v_JNK_, v_MEKK6_, v_MEK6_, v_STAT_, v_Grb2_, v_Shc_, v_SOS_, v_RB_, v_ELK_, v_BAD_, v_NFKB_, v_RAS_, v_GAB1_, v_PIP3_, v_PI3K_, v_PDK1_, v_PTEN_, v_c-Raf_, v_AKT_,v_LKB1_, v_MEK_, v_GSK3β_, v_AMPK_, v_TSC2_, v_MAPK1,2_, v_RSK_, v_Rheb_, v_mTOR-Raptor_, v_4EBP1_, v_p70S6K_, v_p38_, and v_pS6_.

Protein interaction network motifs (the combination of arcs and transitions): v_EGFR_→v_Grb2_, v_Grb2_→v_Shc_, v_Shc_→v_SOS_, v_SOS_→v_RAS_, v_Grb2_→v_GAB1_, v_GAB1_→v_PI3K_, v_EGFR_→v_SRC_, v_SRC_→v_STAT_, v_PI3K_→v_PIP3_, v_PIP3_→v_PDK1_, v_RAS_→v_c-Raf_, v_PDK1_→v_AKT_, v_RAS_→v_Rac_, v_Rac_→v_MEKK4_, v_MEKK4_→v_MEK4_, v_MEK4_→v_JNK_, v_JNK_→v_STAT_, v_Rac_→v_MEKK6_, v_MEKK6_→v_MEK6_, v_MEK6_→v_p38_, v_p38_→v_STAT_, v_PDK1_→v_p70S6K_, v_PTEN_⊣v_AKT_, v_AKT_⊣v_c-Raf_, v_AKT_⊣v_GSK3β_, v_AKT_⊣v_TSC2_, v_AKT_⊣v_AMPK_, v_AKT_⊣v_BAD_, v_AKT_→v_NFKB_, v_AKT_→v_p70S6K_, v_LKB1_→v_AMPK_, v_MEK_→v_MAPK1,2_, v_MAPK1,2_→v_RB_, v_MAPK1,2_→v_ELK_, v_MAPK1,2_→v_STAT_, v_GSK3β_→v_TSC2_, v_AMPK_→v_TSC2_, v_MAPK1,2_⊣v_EGFR_, v_MAPK1,2_⊣v_TSC2_, v_MAPK1,2_→v_p70S6K_, v_MAPK1,2_→v_RSK_, v_RSK_⊣v_TSC2_, v_TSC2_⊣v_Rheb_, v_Rheb_→v_mTOR-Raptor_, v_AKT_→v_mTOR-Raptor_, v_mTOR-Raptor_→v_4EBP1_, v_mTOR-Raptor_→v_p70S6K_, v_p70S6K_⊣v_EGFR_, v_SRC_⊣v_SRC_, v_Rac_⊣v_Rac_, v_MEKK4_⊣v_MEKK4_, v_MEK4_⊣v_MEK4_, v_JNK_⊣v_JNK_, v_MEKK6_⊣v_MEKK6_, v_MEK6_⊣v_MEK6_, v_STAT_⊣v_STAT_, v_Grb2_⊣v_Grb2_, v_Shc_⊣v_Shc_, v_SOS_⊣v_SOS_, v_RAS_⊣v_RAS_, v_c-Raf_⊣v_c-Raf_, v_MEK_⊣v_MEK_, v_MAPK1,2_⊣v_MAPK1,2_, v_RB_⊣v_RB_, v_ELK_⊣v_ELK_, v_RSK_⊣v_RSK_, v_GAB1_⊣v_GAB1_, v_PIP3_⊣v_PIP3_, v_p38_⊣v_p38, _v_PI3K_⊣v_PI3K_, v_PDK1_⊣v_PDK1_, v_AKT_⊣v_AKT_, v_BAD_⊣v_BAD_, v_NFKB_⊣v_NFKB_, v_AMPK_⊣v_AMPK_, v_mTOR-Raptor_⊣v_mTOR-Raptor_, v_p70S6K_⊣v_p70S6K_, v_pS6_⊣v_pS6_, v_4EBP1_⊣v_4EBP1_.

Notice that the last several edges are self-inhibitory loops (e.g., v_Ras_⊣v_Ras_). These loops are used to model regulatory mechanisms that are not present in the model network.

For molecules that do not have specific inhibitory edges modeled in the network, we use the self-inhibitory loop to prevent exponential increase in the token counts and to model inhibitory mechanisms beyond the scope of the network. For example, consider the molecule Ras in the network shown in Figure 1. In the model, this protein is not inhibited. However, biologically we know that Ras has intrinsic GTPase function which inactivate itself. In order to model this, we introduce a self-inhibitory loop.

The differences between the two cell-specific networks are captured by following activity assignments to various proteins in the SPN. In the MDA231 cell line, H^MB^ = {v_Ras_, v_EGF_} and L^MB^ = Ø. In the BT549 cell line, H^BT^ = {v_EGF_} and L^BT^ = {v_PTEN_}.

Of the two perturbations we considered, one significantly knocked down the activity-level of TSC2 and the other knocked down mTOR-Raptor. While the core SPN still modeled these networks, separate *perturbed* activity-assignments were required for each cell line-perturbation pairing: L^MB-TSC2^ = L^MB^∪{v_TSC2_}, L^MB-mTOR^ = L^MB^∪{v_mTOR-Raptor_}, L^BT-TSC2^ = L^BT^∪{v_TSC2_} and L^BT-mTOR^ = L^BT^∪{v_mTOR-Raptor_}.

### Setup for Perturbation Experiments

#### Cell culture and stimulation

Human MDA-MB-231 (MDA231) and BT549 breast cancer cells were routinely maintained in RPMI supplemented with 10% FBS. For signaling experiments, logarithmically growing cells were serum-starved for 16 hours and then subjected to treatments by epidermal growth factor (EGF) (20 ng/mL) (Cell Signaling Technology, Beverly, Massachusetts) for 30 minutes. Controls were incubated for corresponding times with DMSO. To knock down TSC2, cells were treated with short interfering RNA (siRNA) (Dharmacon, Lafayette, Colorado) for 72 hours prior to EGF stimulation. Control cells were transfected with non-targeting (N/T) siRNA (Dharmacon, Lafayette, Colorado) prior to EGF treatment.

#### Antibodies

The following antibodies were used for immunoblotting: anti-phospho-p44/42 MAPK, anti-phospho-GSK3β (S21/S9); anti-phospho-AKT(ser473); anti-phospho-TSC2(T1462); anti-phospho-mTOR(S2448); anti-phospho-P70S6K(T389) (Cell Signaling Technology, Boston, Massachusetts); and anti-β-Actin (Sigma-Aldrich, St. Louis, Missouri).

#### SDS-PAGE and immunoblotting

Cells were lysed by incubation on ice for 15 minutes in a sample lysis buffer (50 mM Hepes, 150 mM NaCl, 1 mM EGTA, 10 mM Sodium Pyrophosphate, pH 7.4, 100 nM NaF, 1.5 mM MgCl2, 10% glycerol, 1% Triton X-100 plus protease inhibitors; aprotinin, bestatin, leupeptin, E-64, and pepstatin A). Cell lysates were centrifuged at 15,000 g for 20 minutes at 4°C. The supernatant was frozen and stored at −20°C. Protein concentrations were determined using a protein-assay system (BCA, Bio-Rad, Hercules, California), with BSA as a standard. For immunoblotting, proteins (25 µg) were separated by SDS-PAGE and transferred to Hybond-C membrane (GE Healthcare, Piscataway, New Jersey). Blots were blocked for 60 minutes and incubated with primary antibodies overnight, followed by goat anti-mouse IgG-HRP (1∶30,000; Cell Signaling Technology, Boston, Massachusetts) or goat anti-rabbit IgG-HRP (1∶10,000; Cell Signaling Technology) for 1 hour. Secondary antibodies were detected by enhanced chemiluminescence (ECL) reagent (GE Healthcare, Piscataway, New Jersey). All experiments were repeated a minimum of three independent times.

#### Setup for perturbation simulations

To select the block duration parameter, B, we compared the experimentally derived fold change of AKT in the MDA231 cell line to the AKT fold changes predicted for B = 10, 20, 50, 100, and 1000. We found B = 20 to be the best fit and used this value for all simulations in this study.

We also experimented with input parameter r, the numbers of individual simulation runs averaged per simulation. We tried a range extending from *r* = 100 to *r* = 1000. We found that no observable changes occurred in trends for *r*≥400. Therefore, *r* = 400 was used for all simulations in this study.

We considered both the zero and experimentally derived initial states as the initial markings for the TSC inhibition simulations. The experimental states for both cell lines were derived from western blots produced from cells that were incubated in DMSO and serum-starved for 16 hours. Unsampled molecules were assigned a marking of zero. The number of tokens assigned to each sampled molecule was directly proportional to the darkness of the line on the western blot. This assignment was done by hand, though devising automated and standardized methods for the construction of experimentally derived initial states is an important direction for future work. Since most of the molecules in the network were not sampled, only mTOR-Raptor, TSC2, GSK3β, p70S6K, AKT, and MAPK were given non-zero markings. The initial markings used are shown in Table 1.

**Table 1:** Experimentally Derived Initial Markings Used in the Simulations.

Molecule	*MB231*		*BT549*	
	Control	TSC2 Inhibited	Control	TSC2 Inhibited
mTOR-Raptor	0	1	5	5
TSC2	0	0	6	0
GSK3β	5	3	3	6
p70S6K	0	2	0	0
AKT	0	0	7	7
MAPK	2	6	1	2

Since experimental results for the mTOR-Raptor inhibition were obtained from literature, we did not have experimental results for construction of experimentally derived initial states. Therefore, we used the zero initial states for the mTOR-Raptor inhibition simulations.

## Results

In order to evaluate the accuracy of our simulation method, we tested its predictions of the effect of targeted manipulations on two cell-specific versions of the signaling network depicted in Figure 1. In each cell line, a TSC2-specific siRNA was applied and the concentration of several key proteins in the EGFR network were sampled 30 minutes after stimulation with EGF. This was repeated in the absence of the TSC2 siRNA in order to obtain the concentration in the control network. We also collected a corpus of literature detailing the response of signaling proteins activity-levels to the inhibition of mTOR-Raptor using Rapamyacin [43],[58]. Predictions were generated by our simulator for the TSC2 and mTOR-Raptor perturbations in both cell lines.

### Simulation

To simulate a perturbation, we used two networks both based on the signaling network shown in Figure 1: the control network for the cell line and the perturbed network for the cell line. The control networks for the cell lines were different because it was important to model the cell-specific mutations. In the case of the BT549 cell line, there is a mutation that leads to the loss of PTEN, which makes AKT always active. In the MDA231 cell line, there is a mutation in Ras, which makes it always active. As shown in the formulation of the model, these are modeled using fixed activity assignments in the simulator.

**Figure 9:**
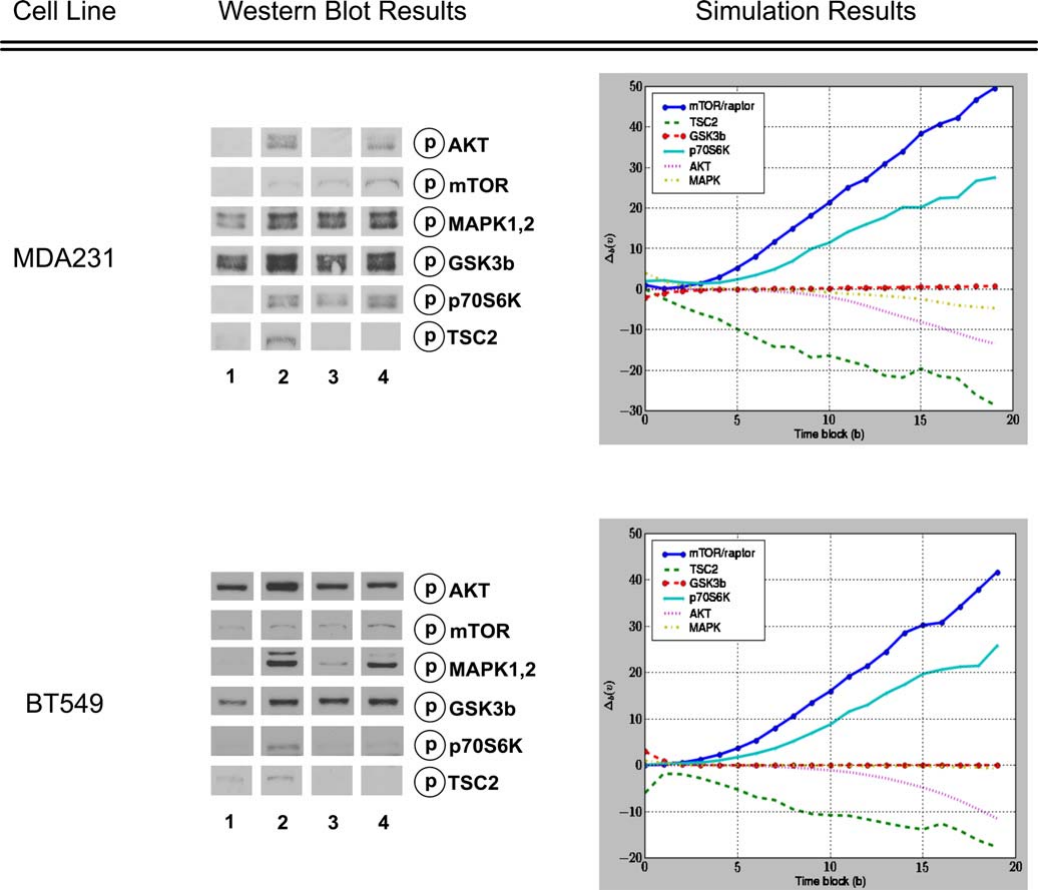
The Results of the TSC2 Perturbation Experiments and Simulations. In the western blots, columns (or lanes) are as follows: (1) non-targeting (NT) control siRNA, (2) NT siRNA+EGF, (3) TSC2 siRNA, (4) TSC2 siRNA+EGF. The effect of the TSC2 siRNA on a given molecule can be assessed by comparing column 4 against column 2. For each molecule in the western blot, there is a corresponding simulation curve showing the predicted change in protein activity over time. For the purposes of this analysis, we compared the concentration change after 20 time steps (the left-most data points in the plots) for each molecule. Each simulation point corresponds to the average of 400 measurements that were computed using the procedure described in Figure 8. Experimentally derived initial states were used in the simulations. The results of both the experiments and simulations are qualitatively summarized in Table 3.

**Figure 10:**
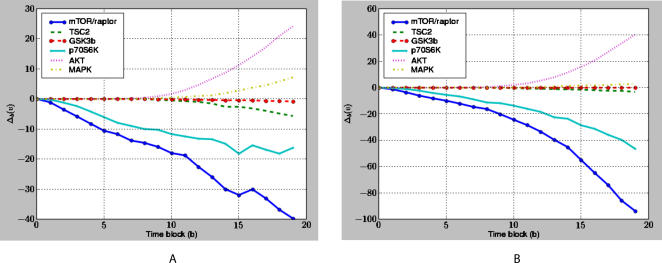
The Predicted Response of the Network to an mTOR-Raptor Perturbation. The predicted response of the network to a mTOR-Raptor perturbation in the (A) MDA231 and (B) BT549 cell lines. Our method predicts that the amount of available AKT increases in response to the perturbation, which is in agreement with results published in the literature [43],[58]. Our method also predicts that the activity-level of p70S6K in the MDA231 cell line decreases in response to the perturbation, which has been observed experimentally [59]. Each point corresponds to the average of 400 measurements that were computed using the procedure described in Figure 8.

The TSC2 (mTOR-Raptor) perturbed network for a cell line was created by taking the control network and fixing the activity-level of TSC2 (mTOR-Raptor) to zero for the duration of the simulation, effectively simulating the pharmacological inhibition of the protein. For each cell-line/perturbation pair, we ran the simulator on the control and perturbed networks using the DifferentialSimulate procedure in Figure 8 which computed the change in token-counts induced by the perturbation for all proteins in the model. These change plots are shown in Figure 9 for TSC2 and in Figure 10 for mTOR-Raptor. We ran the simulations using both experimentally derived initial states as well as zero initial states. The initial state used did not change the overall trends observed in the simulations.

Using the t-test described in the Methods section, we also computed the statistical significance of the final time block (b = 20) for each molecule considered. For each molecule considered, 400 runs, 20 time blocks, and 50 samples were used. With the exception of GSK3β which did not show a significant response to the perturbation, the changes of all other proteins sampled were beyond the 0.05 significance level (see Table 2). The statistical insignificance of the change in GSK3β is not surprising since, as shown in Figure 1, GSK3β is solely activated by LKB, a molecule fixed high in both cell lines. Thus, we should not expect either perturbation to have a significant effect on the activity of GSK3β, which is what the t-value indicates.

**Table 2:** The T-Values for the Molecules Sampled in the Microarray.

Molecule	t-Value in MDA231	t-Value in BT549
mTOR-Raptor	41.72	30.53
TSC2	21.65	8.28
GSK3β	0.42	0.10
p70S6K	14.22	5.83
AKT	6.60	9.55
MAPK	16.35	18.93

The critical value for an alpha value of 0.05 with 50 samples is 2.0086. Note that the t-values for all molecules except for GSK3β are larger than this value, confirming that these changes are statistically significantly.

### Experimental Results

After the TSC2 perturbation was applied to a cell line, the protein concentrations were collected using western blots. Details are given in the Materials and Methods section. The western blot results are shown in Figure 9.

## Discussion

As can be seen in Table 3, our method correctly predicted the *relative* protein activity-level changes induced by the TSC2 perturbation in both cell lines, for most molecules sampled. Notice that *no change* (–) was reported for the predicted response of MAPK to the TSC2 perturbation despite the fact that a small change did occur in its marking during the simulation (see Figure 9) and the t-value for the change is significant (see Table 2). At first, interpreting this value as *no change* may seem misleading. However, one of the significant challenges in experimental perturbation experiments is separating true system responses from the background noise created by experimental variables that cannot be precisely controlled (among them cell population sizes, variability in microarray antibody binding effectiveness, and limited sensitivity of hardware and software used to quantify experimental results). As a result, a common practice is to only consider those substantial changes that are well beyond the background noise level. Our interpretation of the small predicted change in MAPK as *no change* reflects the fact that such small changes would not be detectable in microarray or western blot results. Thus, though such a small fluctuation might have occurred in the real data, it would not have been detected by the biologists and most likely would appear in the experimental data to have not changed.

**Table 3:** Summary of the Effect of Perturbation Reported by Experimental and Simulated Methods.

Molecule	MB231		BT549	
	Experiment	Simulation	Experiment	Simulation
mTOR-Raptor	↑	↑	↑ or −	↑
TSC2	↓	↓	↓	↓
GSK3β	−	−	−	−
p70S6K	↑	↑	↓	↑
AKT	↓ or −	↓	↓	↓
MAPK	−	−	−	−

The up arrow (↑) indicates that the perturbation caused a rise in the level of the phosphorylated protein; the straight line (−) indicates no change; and the down arrow (↓) indicates that a decrease occurred. Values in the *Experiment* column were estimated by comparing lanes 4 and 2 in Figure 9. We estimated the *Simulation* column by determining whether the top quartile of the distribution for the final time point was above, below, or at zero. In some cases it is difficult to judge for certain whether the total quantity of the phosphorylated protein changed or remained the same—both for the experimental and computational cases. In these situations, we indicated the uncertainty by listing the possible changes that the protein *could* have feasibly undergone.

Similar reasoning guided our decision to characterize the simulation (and experimental) results as either up (↑), down (↓), or no change (−) in general. Since the amount of protein registered in a microarray or western blot is not always a reliable indicator of the exact amount of protein (or protein form) being measured, biologists are often reluctant to report degrees of increases or decreases—preferring binary observations such as *up* or *down* which are less subject to influence by extraneous experimental conditions. It is true that our simulation method produces precisely quantified increases or decreases which can be taken to indicate degrees of change in response to perturbations. However, as experimental techniques cannot reliably measure degrees of increase or decrease, we judged the binary (up or down) characterization to be a more reliable way of validating our method. Certainly, our method provides additional information of degrees of change and we consider studying the accuracy of these degrees to be an important area for future work.

Our method also correctly predicted the activity-level change of AKT in response to mTOR-Raptor inhibition as reported by a number of studies [43],[58]. Further, our method predicted that, when mTOR-Raptor is inhibited, the level of p70S6K in the MDA231 cell line decreased, which also had been observed experimentally [59].

The only incorrect prediction made by our method was the activity-level change of p70S6K in the BT549 cell line. However, BT549 cells contain an RB mutation [49] which could alter p70S6K phosphorylation [60]. It is a strength of our simulator that the discrepancy between our method's predictions and the experimental results identified a section of the model in which additional connectivity has been found which might account for the difference observed.

The predictions made by our simulator would be exceedingly difficult to derive by visual or manual inspection. Table 4 shows the number of paths between several pairs of compounds within the network. Where there is more than one path connecting two molecules, feed forward and feed backward loops are present. Attempting to determine, by hand, how these different loops will interact with one another is, by itself, a difficult endeavor even when not considering the additional task of deriving the rest of the network dynamics simultaneously. For the larger networks that are now becoming available, computational analysis becomes even more crucial to obtaining insights into the dynamic behavior of the network.

**Table 4:** Number of Paths Connecting Several Pairs of Compounds in the EGFR Model Used in Our Simulations

Source Protein	Destination Protein	Number of Paths
EGFR	TSC2	7
AKT	mTOR-Raptor	6
MEK	EGFR	4
AKT	p70S6K	8

The multiple paths connecting pairs of proteins highlight the complex interactions present within the network that give rise to its overall dynamic behavior.

Despite the complexity of the network dynamics, it was straightforward to find and integrate the connectivity information used to build it. Most of the information sources [36]–[43] established the *existence* of various pathways and provided few or no biochemical or kinetic details. As a result, the literature we used would have provided little assistance is building a parameterized Petri net or ODE model. Due to the proliferation of curated signaling network repositories and searchable literature archives, connectivity information is relatively abundant which makes the ad hoc assembly of networks a relatively straightforward endeavor. This further underscores the advantage of using our method over ODEs or parameterized Petri nets to quickly model and characterize some of the dynamics of a signaling network.

For simulations that will be compared to experimental results, the time parameter must be selected carefully. The time parameter, B, indicates how many time blocks our method will simulate. The time block is an abstract unit of time. Therefore, before comparing experimental results and predictions, it is necessary to determine how many seconds, minutes, or hours correspond to a time block. This can be done by comparing a prediction of the simulator with the experimentally measured activity-level of one or two proteins at several time points in order to determine what time blocks correspond to the different sampled time points. In the present study, we calibrated our time blocks only once for two cell lines and six experimental conditions (two cell lines, with/without TSC2, with/without mTOR-Raptor). To select the time parameter we used the experimentally measured activity changes in two proteins at two time points. In contrast to other predictive dynamic analysis tools which require multiple time points and multiple protein samples in order to calibrate simulation and model parameters, our method has relatively low time and resource investment.

Besides the time parameter, the other component of our simulations which involved experimentally obtained knowledge was the initial states. The experimentally derived initial states require that some experimental data be available providing information on the initial concentrations of individual signaling proteins in the network prior to stimulation. However, in the network that we considered here, the overall behavior of the network and of individual signaling proteins was resilient to changes in the initial states used. Zero and experimentally derived both produced the same overall change predictions. Thus, while experimentally derived initial states may be important for the simulation of some networks, it may well be the case that many networks (such as the one we considered in this paper) can be simulated without this knowledge—further reducing the experimental work that must be done prior to simulation.

The fact that our simulator produced accurate predictions for a variety of experimental conditions using the one core network model and set of simulation parameters also distinguishes our method from other predictive approaches. The only aspects of the model that were modified during the simulations were activity-levels reflecting the immediate effects of either the underlying tumor mutations (Ras and PTEN) or the perturbations (mTOR-Raptor and TSC2 targeted manipulation). In contrast, the accuracy of ODEs and Petri nets predictions are known to be sensitive to small changes to the model. For comparative studies such as the one conducted in this paper, an ODE or parameterized Petri net model might need to be re-constructed with different parameters for each experimental condition of interest. As a result, while it is possible to obtain our simulation results using these models, it remains beyond the capabilities of any existing ODE or parameterized Petri net system to provide insights into the effects of experimental conditions on the dynamic behavior of a signaling network with so little initial time and resource investment.

Though our method's predictions will not be as accurate as the results returned by a correctly parameterized ODE, biologists using our method can derive information about a network's dynamic behavior without having to conduct extensive experimentation and computationally expensive parameter estimation. This novel capability offers scientists the exciting prospect of being able to test hypotheses regarding signal propagation in silico. As a result, by using our method researchers can evaluate a wide array of network responses in order to determine the most promising experiments before even entering the laboratory.
